# A Combined Cold Extrusion for a Drive Shaft: A Parametric Study on Tool Geometry

**DOI:** 10.3390/ma13102244

**Published:** 2020-05-13

**Authors:** Tae-Wan Ku

**Affiliations:** Engineering Research Center of Innovative Technology on Advanced Forming, Pusan National University, Geumjeong-gu, Busan 46241, Korea; longtw@pusan.ac.kr; Tel.: +82-51-510-3130

**Keywords:** combined cold extrusion, tool geometry, parametric investigation, preform, spur gear, internal spline

## Abstract

Parametric investigations related to shoulder angle on tool geometry for a combined cold extrusion of a drive shaft, which consisted of spur gear and internal spline structures, were conducted through three-dimensional FE (finite element) simulations. The drive shaft was required to be about 92.00 mm for the face width of the top land on the spur gear part and roughly 22.70 mm for the groove depth of the internal spline section. AISI 1035 carbon steel material with a diameter of 50.00 mm and a length of 121.00 mm was spheroidized and annealed, then used as the initial billet material. A preform as an intermediate workpiece was adopted to avoid the excessive accumulation of plastic deformation during the combined cold extrusion. Accordingly, the cold forging process involves two extrusion operations such as a forward extrusion and a combined extrusion for the preform and the drive shaft. As the main geometric parameters influencing the dimensional quality and the deformed configuration of the final product, the two shoulder angles of ***θ*_1_** and ***θ*_2_** for the preform forging and the combined extrusion were both considered to be appropriate at 30°, 45°, and 60°, respectively. Using nine geometric parameter combinations, three-dimensional finite element simulations were performed, and these were used to evaluate the deformed features and the geometric compatibilities on the spur gear structure and the internal spline feature. Based on these comparative evaluations using the numerically simulated results, it is shown that the dimensional requirements of the target shape can be satisfied with the shoulder angle combination of (45°, 45°) for (***θ*_1_**, ***θ*****_2_**).

## 1. Introduction

In various cold working processes, a combined forging is classified as an operation that merges similar or different sequences together into a single procedure [1,2,3]; it is aimed at process simplification and lead-time reduction by curtailing the number of processes required to obtain a forged component with a complicated shape [4,5]. Typical examples of the combined forging include backward-backward extrusion, forward-backward extrusion, and upsetting-forward operation [6,7,8,9]. In particular, backward-backward extrusion and forward-backward extrusion are collectively called “combined extrusion”. In order to successfully apply these combined extrusions to real manufacturing processes, an objective evaluation of the geometric appropriateness and accuracy of the tool shape, as well as the mechanical properties of the workpiece material, is necessary [10]. As other process variables affecting the product quality, the relative velocity gradient, and the forging speed during the forging operation, the microstructural characteristics, the metal flow, and the friction characteristics between the billet workpiece and the tool surfaces, among others, can be regarded as the main parameters [11,12,13].

This study introduces a drive shaft, which is a torque-carrying metal component used in industrial hydraulic pumps. This mechanical-structural metal member has a three-dimensional complicated aspect wherein two different functional structures such as a spur gear and an internal spline are merged into the shaft [13,14]. Specifically, the spur gear has a unique configuration of sixteen tooth profiles, while the internal spline has a distinctive shape of an irregular hexadecagonal cross-sectioned deep groove. Considering these external features of the drive shaft used for the hydraulic pump, it can easily be envisaged that it would be difficult to directly extrude the initial cylindrical workpiece to the final configuration without an excessive accumulation of plastic deformation. Therefore, in a number of instances, it is reasonable to adopt a preform as an intermediate workpiece [15,16].

Based on the prior results related to the systematic approach to the process design for a drive shaft [17], in order to substantialize the drive shaft using the combined cold extrusion, parametric investigations focusing on the shoulder angle on each extrusion die were conducted and compliance evaluations on the dimensional specifications were carried out, then the appropriate combination of the process parameters was extracted in this study. Here, two shoulder angles of ***θ*_1_** on the extrusion die for the preform forging and ***θ*_2_** on that for the combined extrusion were selected as the main geometric variables. That is, the practically applicable variables for ***θ*_1_** were assumed to be 30°, 45°, and 60°, and those for ***θ*_2_**were also supposed to be 30°, 45°, and 60°. With nine allowable combinations related to the geometric variables on the extrusion dies, FEM-based numerical simulations were performed to investigate the effects of the geometric variables on the product quality and the dimensional compliance. A series of geometric comparisons between the designed drive shaft and the simulated results were conducted with respect to the deep groove within the internal spline having the irregular hexadecagonal cross-section, as well as the sixteen tooth profiles of the spur gear. The results indicated that the drive shaft was well deformed and that the required specifications were closely satisfied when the parameter combination set of (***θ*_1_**, ***θ*_2_**) was (45°, 45°). As a brief summary, the results showed that preform forging and combined extrusion could successfully be adopted to visualize the drive shaft used for the hydraulic pumps with a good consistency regarding the dimensional requirements.

## 2. Drive Shaft with Spur Gear and Internal Spline

### 2.1. Drive Shaft and Combined Extrusion

One of the most important factors in manufacturing a drive shaft with a high degree of geometric difficulty is the reasonable selection of a suitable forging process. Based on the required product characteristics, a thermal working condition for hot and warm and cold forging processes must be selected and the specific forging process for realizing the target shape also has to be defined and applied. In view of these, the spur gears are typified to include many teeth and profiles on a circumferential surface of a round shaft; the internal splines are identified with several grooves or ridges in the shaft [13,18].

In terms of mechanical robustness and structural integrity, the metal forged components must satisfy certain crucial characteristics such as smooth tooth faces to secure a sufficient contact surface, a precise gear profile to transmit fitly the external torque into the mechanical member, a high wear resistance against friction and contact behaviors, a high mechanical-structural strength to endure the torsional load induced by the external torque, and a high reliability and durability [19]. From these perspectives, the cold working process was used to produce the drive shaft as the torque-carrying metal member used for the hydraulic pumps. On the other hand, regarding the selection of the appropriate specific forging process, the spur gear geometry can be obtained by the forward extrusion operation, while the internal spline can be achieved using the backward extrusion procedure. However, since the spur gear area and the internal spline region are located nearly adjacent to each other on the drive shaft, severe plastic deformation and unpredictable forging defects can occur and accumulate when both of their geometries are deformed by each separate sequence. Accordingly, a combined extrusion referred to as the bi-directional (forward-backward) cold forging procedure, in which forward extrusion for configuring the spur gear and backward extrusion for realizing the internal spline were merged into a single forging procedure, was proposed in this study.

**Figure 1:**
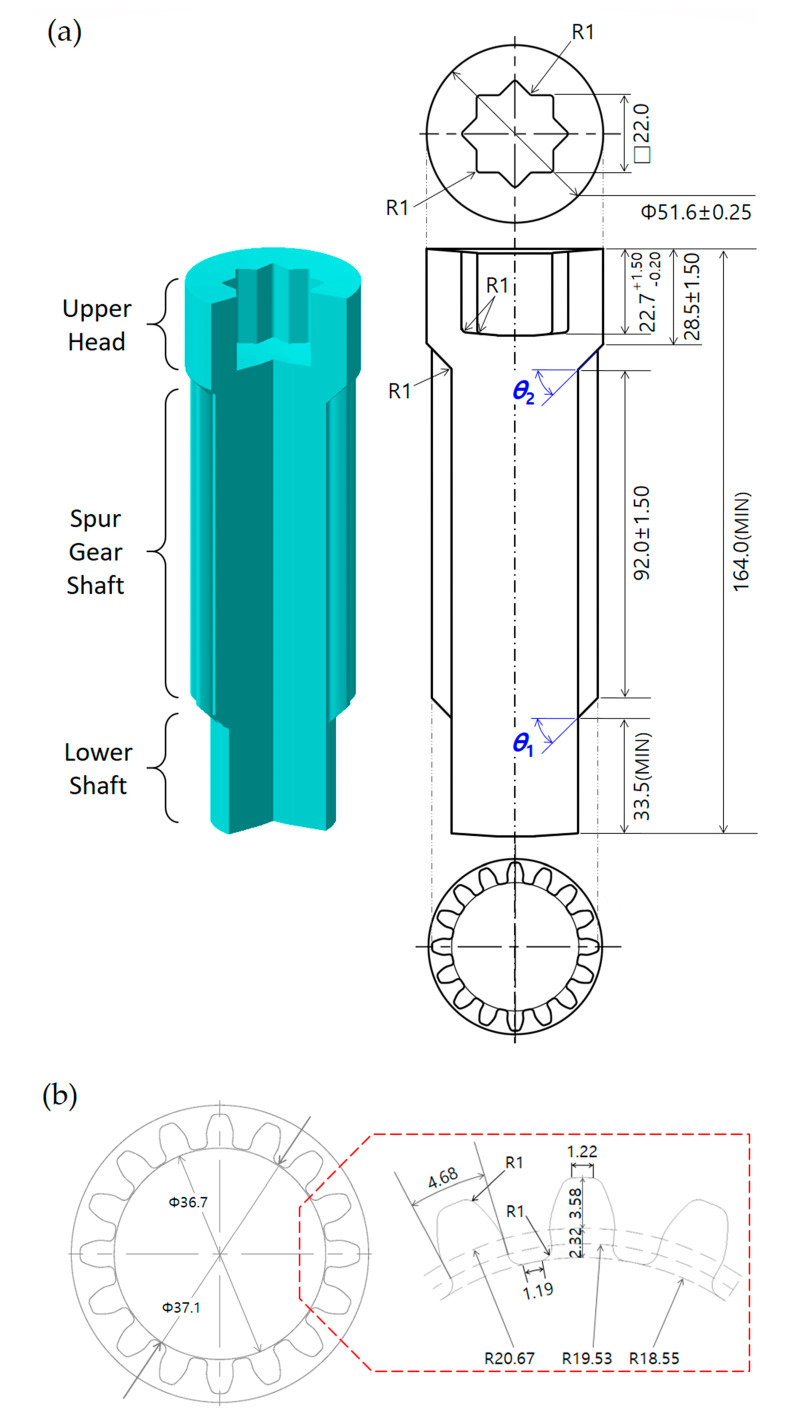
Schematic views and layout of the drive shaft (unit: mm): (**a**) three-dimensional configuration and layout of the drive shaft; (**b**) cross-sectional layout of the spur gear.

Figure 1a illustrates the three-dimensional configuration and two-dimensional layout of the drive shaft that can satisfy these aforementioned requirements. In Figure 1a, it can be seen that the drive shaft has an internal spline structure with a groove depth of 22.7 mm (in an error range of −0.20 mm to 1.50 mm), an outer height of 28.5 mm ± 1.50 mm, and a spur gear geometry with a tooth projecting length (that is, the face width on the top land) of 92.0 mm ± 1.50 mm along the shaft, as well as an extruded length of 33.5 mm on the lower shaft part. Additionally, Figure 1a shows the detailed feature of the internal spline structure, and Figure 1b depicts the specific profiles of the sixteen-tooth spur gear. Regarding the tooth specifications of the spur gear geometry, the addendum and dedendum depths are 3.58 and 2.32 mm, the face and tooth thicknesses are 1.22 mm and 4.68 mm, and the diameter of the bottom land is 37.10 mm ± 0.25 mm.

### 2.2. Preform and Initial Billet

The combined cold extrusion process investigated in this study consisted of the forward and the backward extrusions. Here, the forward extrusion was defined as the actualization of the sixteen-tooth spur gear, while the backward one was defined as realizing the internal spline with the irregular hexadecagonal cross-sectional configuration. However, when the cylindrical-type initial billet is directly deformed to the final target configuration as illustrated in Figure 1, it can lead to an excessive accumulation of plastic deformation and internal damage within the cold-forged workpiece as well as micro-cracks and surface defects. The use of a preform as an intermediate workpiece has been widely adopted as a universal and useful method for solving these difficulties [9,15,16].

**Figure 2:**
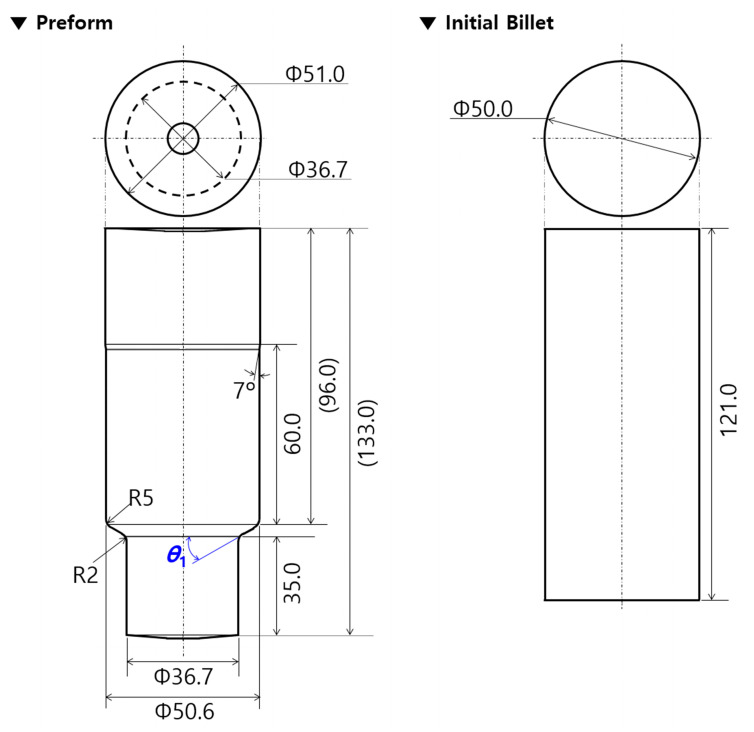
Schematic illustrations of the preform and initial billet for the drive shaft (unit: mm).

Before designing the preform for obtaining the drive shaft, the geometric feature of the lower shaft shown in Figure 1a was first reviewed. Then, it was observed that the forward operation in the combined extrusion makes it impossible to form a shape other than the teeth profiles, and that the detailed features of the lower shaft and the shoulder angle (***θ*_1_**) must be induced from the preform. In consideration of these facts, the preform was designed as shown in Figure 2. In this process, the lower shaft of the preform was outlined with a diameter of 36.7 mm and a length of 35.0 mm, while the upper shaft was designed with a diameter of 51.0 mm and a length of about 96.0 mm in order to provide sufficient workpiece volume for the upper head part and the spur gear section, as shown in Figure 1a. Next, the initial workpiece was also designed using the proposed preform. That is, the lower shaft of the preform was regarded to be forward extruded using the initial round workpiece, and the upper shaft was set to be almost the same as the initial billet. The initial round billet was determined to have a diameter of 50.0 mm and a length of 121.0 mm, as shown in Figure 2. Consequently, the cold forging process consisted of the forward extrusion for the intermediate workpiece as the preform using the initial round billet and the combined extrusion for the drive shaft using the forward extruded workpiece. In detail, the initial round workpiece was first compressed until the forward extruded length reached 35.0 mm. Then, this preform was forged until the groove depth of the internal spline region was roughly 22.7 mm and the extruded face width of the spur gear part reached about 92.0 mm, as shown in Figure 1.

### 2.3. Material Selection and Mechanical Properties

As a mechanical-structural member of the industrial hydraulic pump, the drive shaft must provide a high mechanical and structural strength against torque, high corrosion, and wear resistances, as well as high endurance characteristics, without any inherent defects such as voids or micro-cracks. In various mechanical-structural ferrous metals, the appropriate selection of a workpiece material is necessary to satisfy the requirements listed above. According to these considerations, an AISI 1035 (JIS-G4051-S35C) cold-drawn round bar with a diameter of 50.0 mm, which is widely applied to the shaft, gear, bolt, sprocket, and so forth, was chosen as the raw billet material.

Because the forward extrusion and combined extrusion are successively conducted and the whole extrusion length is quite long, the workpiece material must ensure forgeability and ductility. In many applications of the cold forging process, the raw billet materials have been annealed to soften the yield and the ultimate strengths as well as improve the elongation characteristics [9,10,19,20]. Accordingly, the spheroidizing heat treatment for the AISI 1035 raw material was performed at a temperature of 765 °C ± 10 °C for eight hours and the annealing at a temperature of 695 °C ± 10 °C for eight hours. In order to evaluate the tensile properties, uni-axial tensile tests were conducted using the raw and spheroidizing annealed AISI 1035 specimens. The test specimens were prepared in accordance with the standard specification of ASTM E8/E8M, and a constant test speed of 6.00 mm/min was applied. In addition, the compressive properties of the billet material were tested to establish a guideline for the compressive deformation behavior and verify the compatibility of the compressive stress level. With the raw and annealed AISI 1035 materials, the cylindrical specimens were prepared per ASTM E9-09 standard specification with a diameter of 8.00 mm and a height of 6.40 mm.

**Figure 3:**
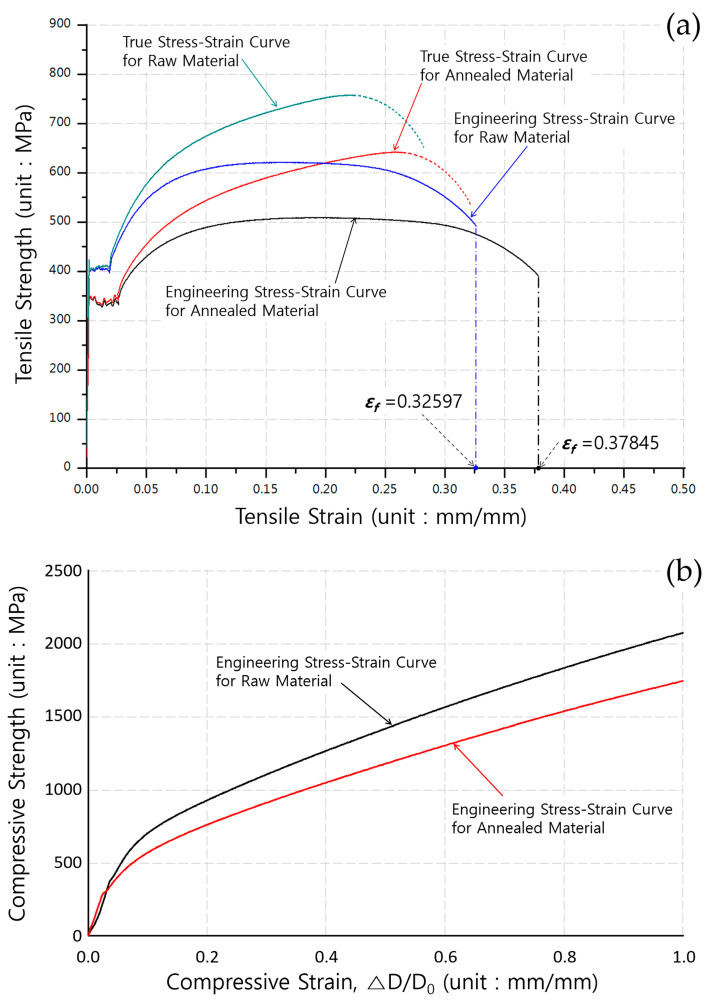
Stress–strain curves of AISI 1035 carbon steel before and after the spheroidizing heat treatment: (**a**) tensile stress–strain curves; (**b**) compressive stress–strain curves.

The tensile properties of AISI 1035 carbon steel, which were obtained from the uni-axial tensile tests, are summarized in Table 1. Furthermore, the stress and strain curves obtained from the uni-axial tensile and compression tests are displayed in Figure 3. The engineering and true stress–strain curves extracted from the uni-axial tensile tests are presented in Figure 3a; these indicate that the tensile yield strength decreased by about 60 MPa and that the ultimate stress was reduced by approximately 110 MPa. Moreover, in the true stress–strain modes, the elongation property was improved by a value of broadly 13.76%. Figure 3b illustrates the compressive stress–strain characteristics of the raw and the annealed AISI 1035 carbon steel materials, and these indicate that the compressive strengths of the billet materials before and after the annealing were sufficiently gained. Further, the compressive yield stress and the ultimate strength of the heat-treated specimen were slightly decreased, but the degrees of those compressive strengths are shown to be sufficient.

## 3. Geometric Parameters and FE Simulation Models

### 3.1. Geometric Parameters and Tool Structures

As shown in Figure 1a, the geometric parameters of the drive shaft were determined as both the shoulder angles of ***θ*_1_** and ***θ*_2_**. Because the drive shaft has disparate structures such as the spur gear and the internal spline, combined with the fact that the forged product quality is more influenced than that derived from other process parameters, the shoulder angles were selected as the actually applicable parameters for simultaneously obtaining the forward extruded tooth profile and the backward extruded deep groove by combined extrusion. The cold forging process is generally regarded to be a quasi-static process, so the influence of the strain rate is typically ignored. In view of this, a series of elastic recovery behaviors of the workpiece material during cold forging can already be neglected, so the dimensional variation can be disregarded as well. This means that the geometries of the drive shaft eventually match the tool configurations.

**Figure 4:**
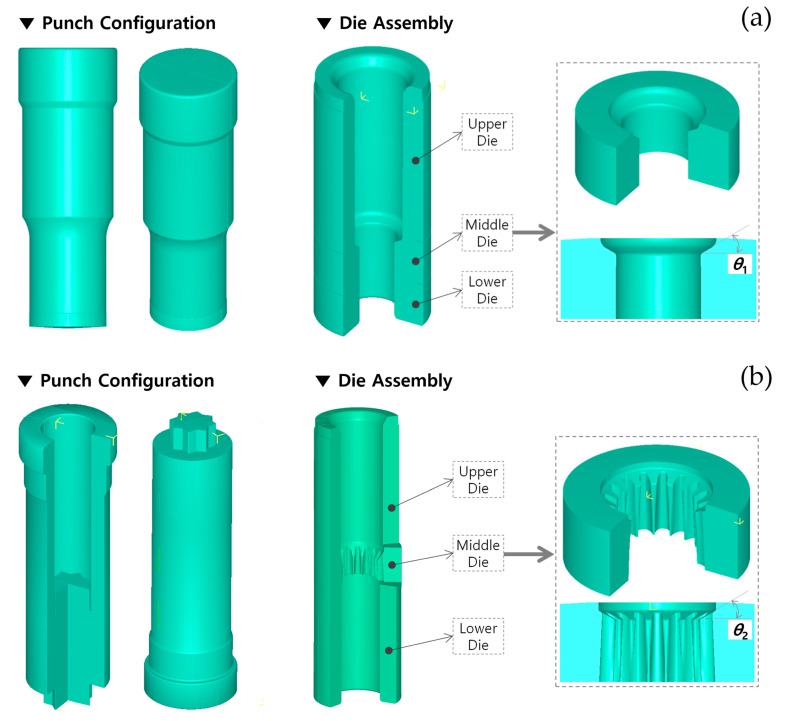
Tool configuration and geometric parameters on each middle die for producing the preform and drive shaft: (**a**) shoulder angle on the middle die for the preform extrusion; (**b**) shoulder angle on the middle die for the combined extrusion.

Therefore, the geometric variables of the drive shaft can be identically regarded as the shoulder angles on the extrusion dies (middle), as illustrated in Figure 4. In detail, the shoulder angle (***θ*_1_**) in Figure 1a was considered to be induced from the preform, so the slope angle was directly applied to that of the forward extrusion die (that is, the shoulder angle on the middle die), as shown in Figure 4a. In addition, the other angle (***θ*_2_**) in Figure 1a was believed to have resulted from the combined cold extrusion, and this angle was also accepted to that on the middle die for the combined extrusion, as depicted in Figure 4b.

### 3.2. FE Simulation Models

**Figure 5:**
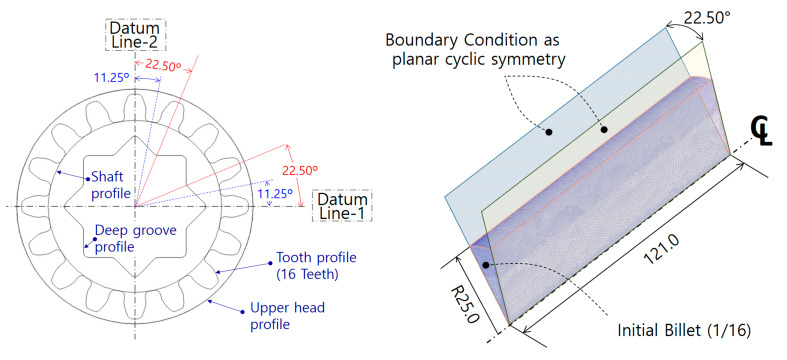
Geometric symmetricity and boundary conditions.

As the cold forging process consisted of the forward and combined extrusion, these separated forging operations were necessarily both individual FEM-based numerical simulation models. In other words, two FE models must be constructed, such as one related to the forward forging process for the preform and the other related to the combined extrusion operation for the drive shaft. Figure 5 shows the superimposed cross-sectional profiles of the lower shaft part and the spur gear section, as well as the internal spline region, which were all positioned at the same central axis. As both the datum lines were orthogonal to each other, the internal spline of the upper head section was presented as having cyclic planar symmetric characteristics with a unit angle of 22.50°, and the sixteen-tooth spur gear part was illustrated to have a structure with a unit angle of 11.25°. These results indicate that the FE models for the upper head and the spur gear parts were successfully constructed, with three-dimensional one-sixteenth (1/16) and one-thirty-second (1/32) structures, respectively. However, because the combined extrusion process should be simultaneously operated, it was revealed that the numerical simulation models are suitable for adopting the three-dimensional one-sixteenth (1/16) FE models derived from the cyclic planar symmetric condition. Thus, the three-dimensional numerical simulation related to the forward extrusion for configuring the preform from the round billet was also proper for application to the one-sixteenth (1/16) structure. Based on these considerations regarding the geometric symmetricity and the boundary condition, each FE model required for the cold forging simulations was set-up according to the minimized one-sixteenth (1/16) cyclic planar symmetric condition in order to reduce the computation duration.

**Figure 6:**
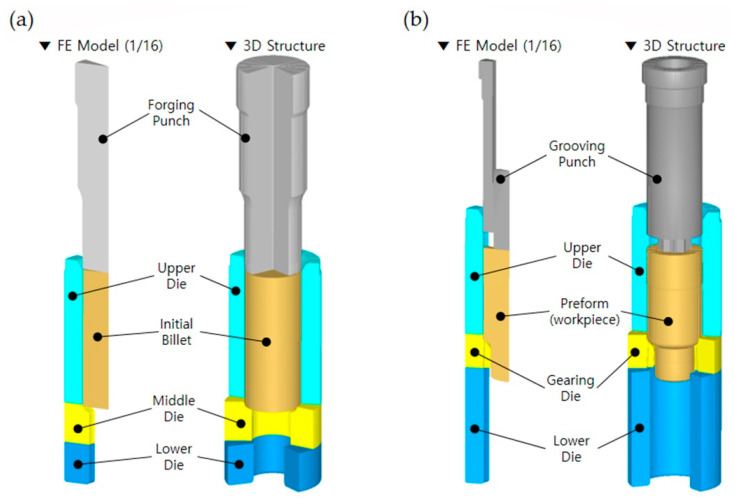
Numerical simulation models for the preform forging and combined extrusion: (**a**) FE model for the forward extrusion model; (**b**) FE model for the combined extrusion.

The numerical simulation model for the forward extrusion to realize the preform is displayed in Figure 6a; it consisted of a forging punch, an upper die, a middle die (that is, an extrusion die), a lower die, and an initial billet of AISI 1035 medium carbon steel with a diameter of 50.0 mm and a height of 121.0 mm, as shown in Figure 2. Furthermore, the FE model for the combined extrusion to produce the final target configuration is also illustrated in Figure 6b, and it was composed of a grooving punch, an upper die, a middle die (that is, an extrusion die), a lower die, and a preform. All of the tool components were regarded as rigid bodies without heat effects during the cold working [21]. The initial workpiece for the forward extrusion and the preform as the intermediate workpiece for the combined extrusion were each regarded to have plastic deformation behavior due to the fact that the elastic recovery of the forged workpiece is typically ignored.

The friction characteristics on the contact interfaces between the forging tool components and the workpiece were considered as the shear friction behavior with a coefficient of 0.098, and the workpiece was coated with a phosphophyllite coating [9,10,19,21]. The numerical simulations were conducted using DEFORM-3D^TM^, and as these procedures were defined as quasi-static processes, the effect of the strain rate was neglected. In order to investigate and verify the effects of the geometric parameters on the product quality and the dimensional compliance, the practically applicable variables for ***θ*_1_** were assumed to be 30°, 45°, and 60°, and those for ***θ*_2_** were also supposed to be 30°, 45°, and 60°. Because there was obviously expected to be an interrelationship between these process variables, a series of parametric investigations with various combinations of the shoulder angles were pursued, and the results are summarized in Table 2.

## 4. FEM-Based Numerical Simulations

### 4.1. Preform Forging Simulations by Forward Extrusion

**Figure 7:**
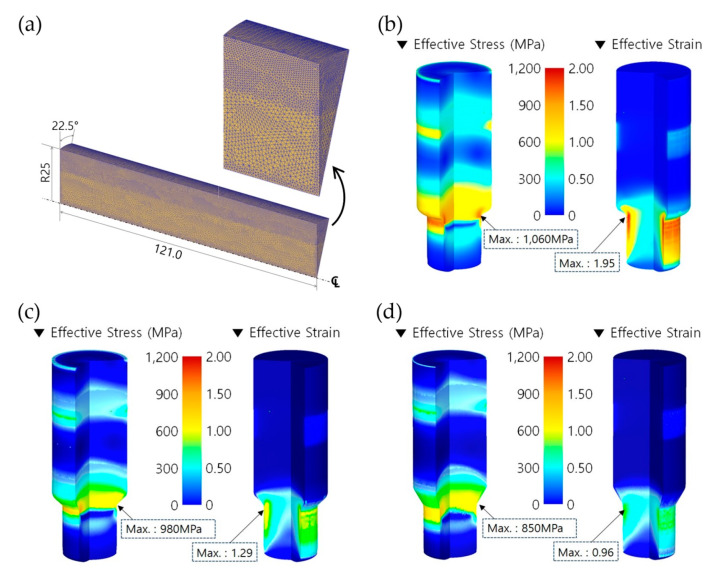
(**a**) Initial mesh structure with about 260,000 tetrahedral elements (unit: mm); (**b**) effective stress and strain distribution with a ***θ*_1_** of 30°; (**c**) effective stress and strain distribution with a ***θ*_1_** of 45°; (**d**) effective stress and strain distribution with a ***θ*_1_** of 60°.

The numerical simulation of the forward extrusion was first carried out in order to obtain the preform using the spheroidizing annealed AISI 1035 workpiece with a diameter of 50.00 mm and a height of 121.00 mm. The forward extrusion process was defined as having the one-sixteenth cyclic planar symmetric condition, as illustrated in Figure 6a. The initial billet material was assumed to be a deformable body with plastic deformation behavior, an automatic remeshing scheme was adopted, and the mesh structure of the one-sixteenth FE model was discretized with about 260,000 tetrahedral elements. Figure 7a depicts the discretized mesh structure, which was varied to be coarse in the center part and dense around the outer region of the initial billet. As the shoulder angle (***θ*_1_**) on the middle die for the preform extrusion was selected among the three values of 30°, 45°, and 60°, three forward extrusion simulations using the initial billet in Figure 7a were performed in order to visualize the preform with a forward extruded length of 35.00 mm on the lower shaft, as presented in Figure 2a.

Regarding the results obtained from the forward extrusion simulation with a shoulder angle of 30°, Figure 7b shows the effective stress and strain distributions. A maximum effective stress and strain of about 1060 MPa and 1.95 were predicted at the extruding neck region, and the forward extruded length was satisfied to be roughly 35.00 mm when the punch stroke reached about 27.51 mm. When a slope angle of 45° was applied to the preform, as shown in Figure 7c, the maximum effective stress and the maximum effective strain were found to be approximately 980 MPa and 1.29 around the shoulder part of the preform, and the punch stroke to attain the required forward extrusion length of 35.00 mm was subsequently about 28.73 mm. In addition, Figure 7d depicts the effective stress and strain distributions visualized by the forward extrusion simulation with the shoulder angle of 60°, and the maximum values were observed to be 850 MPa and 0.96. Furthermore, this cold extrusion simulation was completed with a punch stroke of approximately 31.52 mm in order to secure the extrusion length on the lower shaft.

**Figure 8:**
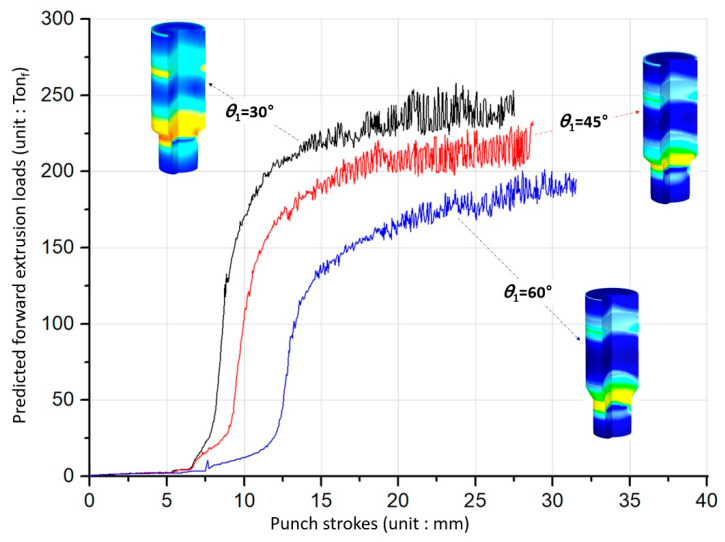
Cold forging load histories for the forward extrusion of the preform.

The simulated results with regards to the forward extrusion for obtaining the preform show that the effective stresses and strains were typically distributed, and that the maximum values were similarly positioned around the extruded neck region of the deformed preforms. In particular, it was observed that the effective stress and strain levels, as well as the maximum values, tended to decrease with an increasing shoulder angle. These facts can be clarified by predicting the forward extrusion loads. Figure 8 shows three forging load histories with the shoulder angle (***θ*_1_**) of the preform being given as 30°, 45°, or 60°. It was then revealed that the cold forging load decreases as the shoulder angle is increased. It suggests that the large shoulder angle leads to a decrease in the cold forging load due to the smoother metal flows around the extrusion die during the forward extrusion operation.

### 4.2. Cold Forging Simulations by Combined Extrusion

This combined extrusion operation for the drive shaft was regarded to be responsible for forming the irregular hexadecagonal deep groove with a depth of about 22.7 mm at the upper head part; it was simultaneously responsible for shaping the sixteen-tooth spur gear structure with an extruded length of roughly 92.0 mm. In order to validate the effects of the geometric parameters on the product quality of the drive shaft, both the shoulder angles of ***θ*_1_** and ***θ*_2_**were predefined as depicted in Figure 1, Figure 2 and Figure 4. As shown in Figure 7, the forward extrusion simulations to visualize the preform were already conducted using the three geometric variables of 30°, 45°, and 60°, because the shoulder angle (***θ*_1_**) was considered to be derived from the preform extrusion. Accordingly, the combined extrusion simulations were performed using the three preforms with different shoulder angles (***θ*_1_**). As summarized in Table 2, because the shoulder angle (***θ*_2_**) on the extrusion die (middle) for shaping the irregular hexadecagonal deep groove and the sixteen-tooth profile of the drive shaft was also chosen among 30°, 45°, and 60°, nine FE models with the same boundary conditions were built up in total.

**Figure 9:**
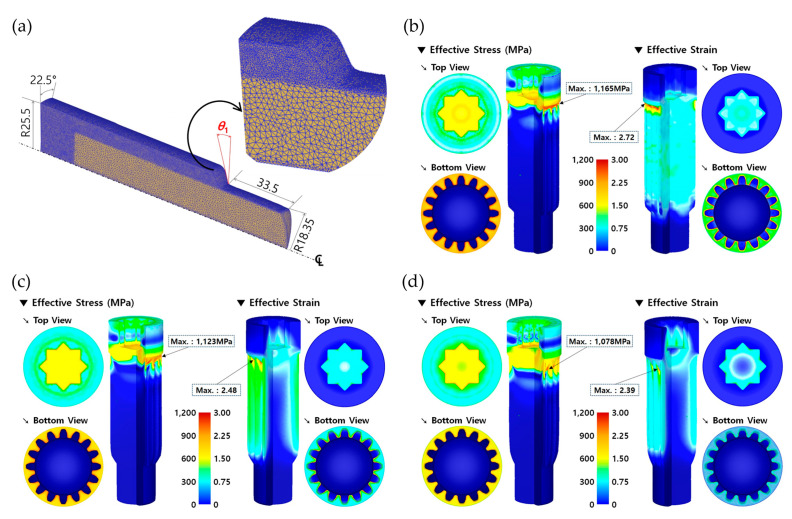
(**a**) Reconstructed mesh structure with about 330,000 tetrahedral elements (unit: mm); (**b**) effective stress and strain distribution with a ***θ*_1_** = 30° and a ***θ*_2_** = 30°; (**c**) effective stress and strain distribution with a ***θ*_1_** = 45° and a ***θ*_2_** = 45°; (**d**) effective stress and strain distribution with a ***θ*_1_** = 60° and a ***θ*_2_** = 60°.

As mentioned previously, the inherent residual stress resulting from the forward extrusion operation in Figure 7 can be eliminated by low temperature annealing for 3 h at 700 °C ± 10 °C and about 30 h at room temperature (about 20 °C). This means that the residual stress within the cold forged preform is recovered to be zero, so the combined extrusion simulation can be simply performed using the deformed layout of the preform. The preforms that the residual stresses were relieved by were re-discretized and re-meshed using roughly 330,000 tetrahedral elements. Figure 9a depicts the discretized mesh structure of the preform, where the mesh density was varied so as to be denser around the outer region in order to precisely simulate and visualize the deep groove and the spur gear configurations. In addition, the cyclic planar symmetric condition was applied as the boundary condition. Using the re-discretized preforms and the FE models provided in Figure 4b and Figure 6b, combined extrusion simulations were carried out. In the nine combinations related to both of the parameters summarized in Table 2, the FEM-based numerical simulation results as the representative combination sets of (***θ*_1_**, ***θ*_2_**), such as (30°, 30°), (45°, 45°), and (60°, 60°), are presented in Figure 9. Here, the shoulder angles of ***θ*_1_** and ***θ*_2_** for the preform and the drive shaft were identified with those on the middle dies for the preform operation and the combined extrusion, respectively. In order to determine whether or not the combined extrusion simulation was completed successfully, the reference length was selected as the backward extruded height of about 28.50 mm on the upper head region because it was hard to gauge the deep groove depth of roughly 22.70 mm.

The effective stress and strain distributions for case 1 with the shoulder angle combination of (30°, 30°) are depicted in Figure 9b. The maximum effective stress and strain of about 1165 MPa and 2.72 were obtained at the extruding neck region, and the backward extruded height was satisfied to be roughly 28.50 mm when the punch stroke reached about 90.95 mm. In case 5, with the shoulder angle set of (45°, 45°) as shown in Figure 9c, the maximum effective stress and strain were measured to be approximately 1,123 MPa and 2.48 around the shoulder part of the drive shaft. Then, the punch stroke to satisfy the reference height of 28.50 mm was measured at about 88.28 mm. In addition, Figure 9d illustrates the effective stress and strain distributions as visualized by the combined extrusion simulation with the geometric parameter set of (60°, 60°); their maximum values were respectively predicted to be 1078 MPa and 2.39. Furthermore, this cold extrusion simulation was completed with the punch stroke of approximately 84.01 mm in order to secure the extrusion length on the lower shaft. Specifically, it was observed that all of the locally concentrated maximum values were similarly positioned at the shoulder area of the extruded drive shafts.

**Figure 10:**
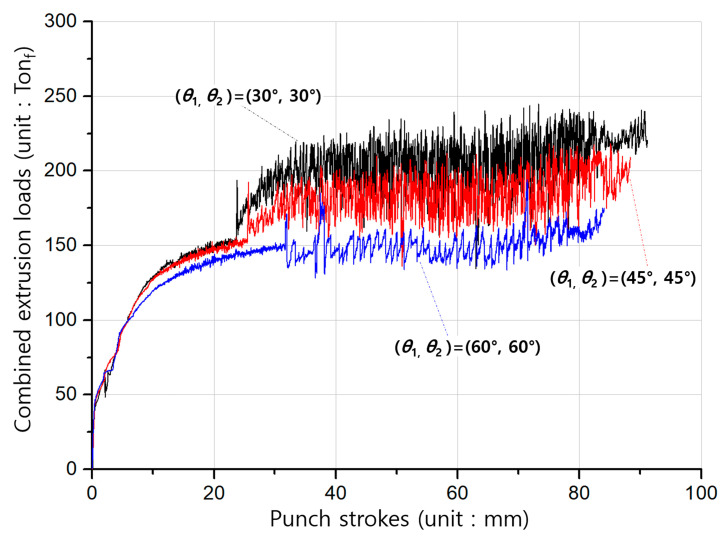
Cold forging load histories for the combined extrusion of the drive shaft.

On the whole, aside from that area, the effective stress and strain were felicitously distributed around the internal spline and the spur gear sections. Moreover, it should be noted in the numerical simulation results that the stress and strain tended to decrease with an increasing shoulder angle (***θ*_2_**), and this was consistent with the results of the forward forging for the preform. These characteristics can also be explained by the forging load properties of the combined extrusion, as shown in Figure 10. That is, the load levels in Figure 10 were revealed to decrease slightly when the shoulder angle of the combined extrusion increased.

## 5. Results and Discussions

### 5.1. Determination of Applicable Geometric Parameters

In this study, the parametric investigations with regards to both shoulder angles of ***θ*_1_** and ***θ*_2_** on the extrusion (middle) dies were conducted as shown in Figure 7 and Figure 9, respectively; it was observed that the deformed geometries are numerically well-simulated and the effective stress and strain are also appropriately distributed. In the parametric study, the most important point of all was to survey and determine the actually applicable combination set. In order to support this consideration, the geometries of the drive shaft extracted from the FEM-based numerical simulations were compared with the reference dimensions illustrated in Figure 1. Because the drive shaft was required to match the upper head height of about 28.50 mm, this value was set as the reference dimension to easy approach. That is, when the reference value was approximately 28.50 mm, due to the fact that the face width at the top land of the spur gear was set to be roughly 92.00 mm ± 1.50 mm, it was evaluated whether or not the extruded face width was satisfied by the combined extrusion process.

**Figure 11:**
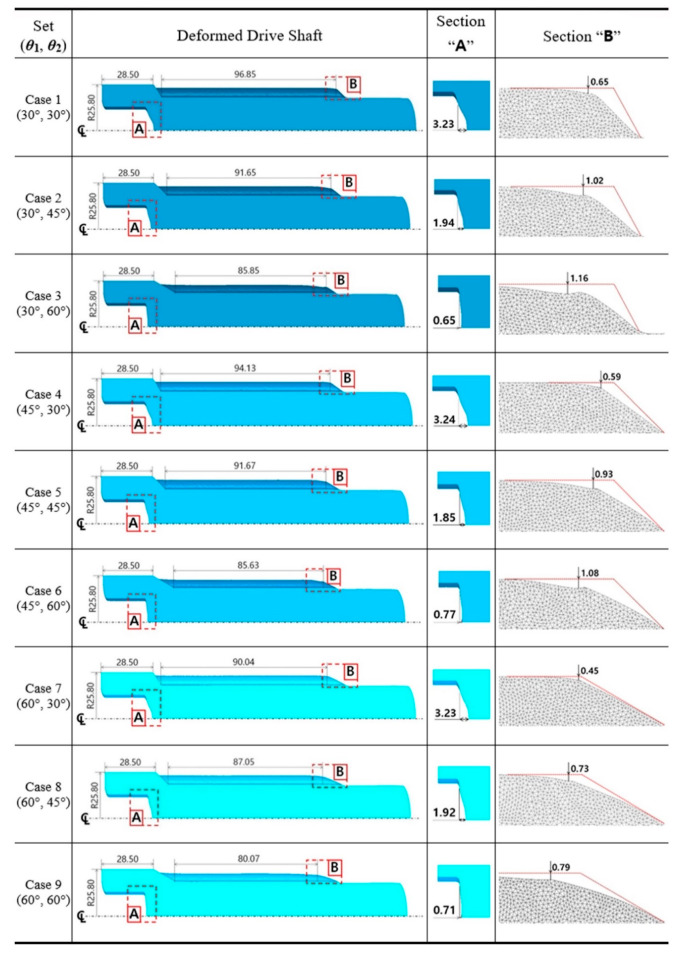
Geometric evaluation of the drive shaft obtained from the combined extrusion (unit: mm).

All of the numerical simulations were conducted with the nine combination sets summarized in Table 2. Figure 11 depicts the deformed layouts and specific dimensions of the drive shaft obtained from the combined extrusion simulations. When the backward extruded height as the reference dimension was about 28.50 mm, the forward extruded face width in the top land was measured for each case; a series of specific deformation behaviors such as the sinking phenomenon along the central axis on the internal spline region and the uneven shape on the extruded tooth end of the spur gear were also evaluated. For the compatibility results with the required face width of roughly 92.00 mm ± 1.50 mm, it was observed that the five parameter sets of (30°, 60°), (45°, 60°), (60°, 30°), (60°, 45°), and (60°, 60°) were inferior to the required width. When the shoulder angle (***θ*_2_**) on the combined extrusion die was also found to be 30°—that is, with the variable sets of (30°, 30°), (45°, 30°), and (60°, 30°)—the sinking depths on the deep grooved upper head part (at the marked section, **A**) were predicted to be roughly 3.23 mm, as large and undesirable values. Regarding the end shape of the extruded tooth, in the cases with the shoulder angle combinations of (30°, 45°), (30°, 60°), and (45°, 60°), the tooth height as the sum of the addendum and the dedendum on the spur gear was unevenly distributed with an error of over 1.00 mm (at the marked section, **B**).

The results derived from these compatibility evaluations were summarized in Table 3. In detail, the numerically predicted results based on design of experiment (DOE) presented in Table 3 show that the small shoulder angle (***θ*_2_** = 30°) tended to induce the severe sinking phenomenon, and the large one (***θ*_2_** = 60°) tended to cause the insufficient face width of the tooth. Further, it is shown that the short face width of the tooth is derived from the large angle (***θ*_1_** = 60°). Ultimately, the most applicable geometric parameters for actualizing the required configuration and dimension provided in Figure 1 were found to be those using the shoulder angle combination of (***θ*_1_** = 45°, ***θ*_2_** =45°).

### 5.2. Process Compatibility of Combined Extrusion

**Figure 12:**
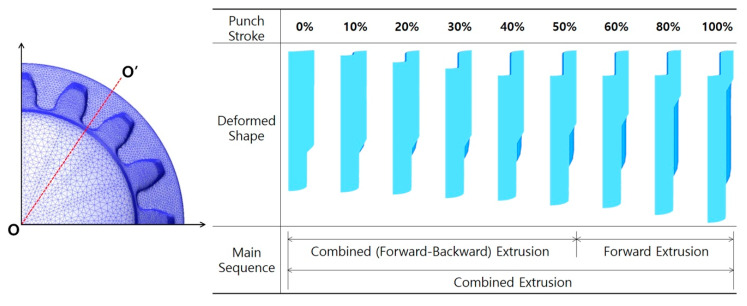
Deformation history and operation sequence of the combined extrusion for the drive shaft.

Because the main geometries of the drive shaft were actually realized by the combined extrusion consisting of the forward extrusion and backward extrusion, the deformation history was traced in terms of the operation sequence of both extrusions in the progress of the cold forging process, when the shoulder angle (***θ*_2_**) of the extrusion die was 45° with the preform obtained by the angle (***θ*_1_**) of 45°. As shown in Figure 12, a cross-section **O**-**O’** on the bottom land of the spur gear geometry was used to trace the deformation history during the combined extrusion. When the preform was compressed by the deep grooving punch, deformed shapes per increments of every 10% based on a total punch stroke of about 88.28 mm—as predicted in Figure 9c and 10—were visualized. The deformation history of the combined extrusion is shown in Figure 12, and it was verified that the backward extrusion for realizing the deep groove at the internal spline region and the forward extrusion for actualizing the sixteen-tooth spur gear were well synchronized as well as sequentially operated during the combined extrusion. It could be seen that the internal spline structure was already formed and that the sixteen-tooth spur gear was partially shaped by the combined extrusion when the punch stroke reached about 40%. Since then, the sixteen-tooth spur gear was mainly forward extruded. In particular, it was revealed that the sinking phenomenon along the central axis on the internal spline region occurred when this combined extrusion was approaching the end sequence of the cold forging. Consequently, it can be summarized that the combined cold extrusion to manufacture the drive shaft with the distinctive shapes of the deep-grooved internal spline structure and the sixteen-tooth spur gear shape was well composed and meaningfully operated with regard to the process compatibility.

### 5.3. Dimensional Relevance

Regarding the results obtained from the numerical investigations on the preform forging and the combined extrusion for realizing the drive shaft, the applicable geometric parameters that can satisfy the required configuration and dimensions, as shown in Figure 1, were regarded to be those with the shoulder angle combination of (***θ*_1_** = 45°, ***θ*_2_** = 45°). Then, the proposed combined extrusion for shaping the drive shaft was also verified to be compatible. In order to investigate the dimensional relevance, the numerically simulated features of the preform and drive shaft were used for comparison with the designed features. That is, the preform obtained from the forward forging simulation shown in Figure 7c was compared with the required dimensions in Figure 2a, while the drive shaft acquired from the combined extrusion simulation presented in Figure 9c was contrasted with the designed dimensions in Figure 1. Image processing software (Geomagic Qualify) visualizing the relative distance difference between both of the comparable features was also used to verify the geometric conformity of the cold-forged metal components in this study.

**Figure 13:**
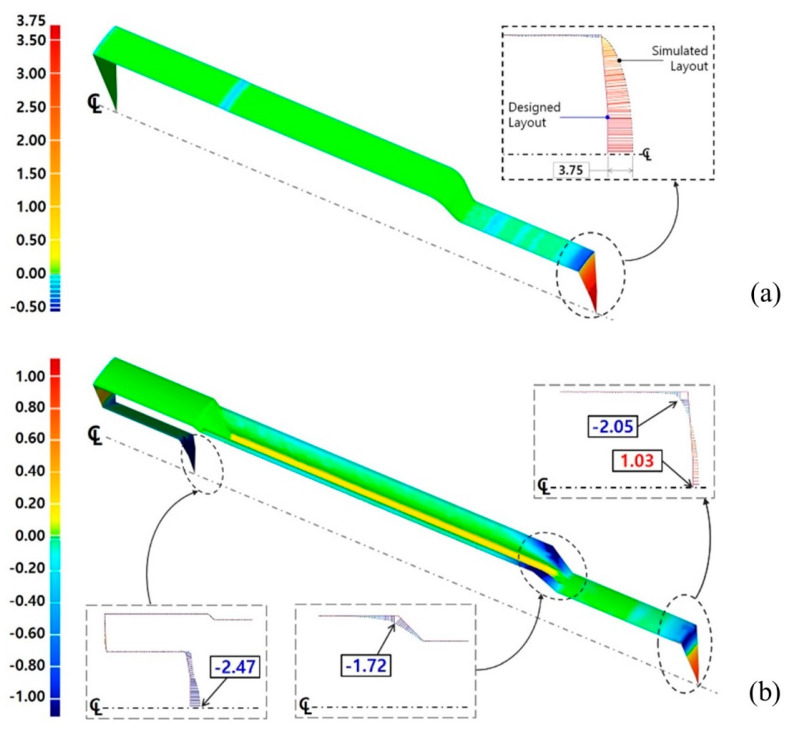
Comparisons of dimensional relevancy between the designed and simulated results (unit: mm): (**a**) relative dimensional error between the simulated and designed preform with a ***θ*_1_** = 45°; (**b**) relative dimensional error between the simulated and designed drive shaft with a ***θ*_1_** = 45° and a ***θ*_2_** = 45°.

Figure 13 shows the compared results with respect to the one-sixteenth cyclic symmetric model of the preform and the drive shaft. Figure 13a presents the result of the comparative investigation between the numerically simulated configuration and the designed target one of the preform. It could be observed from Figure 13a that the dimensional errors were very small and corresponded with the range of −0.25 mm to 0.25 mm. However, the obviously distinctive deformation behavior was shown in the form of excessive protrusion around the end of the lower shaft. The workpiece at the central area was more extruded, relatively, around the external section due to the frictional characteristics between the contact interfaces. For this reason, the numerically predicted preform was more protruded, at nearly 3.75 mm, at the end part on the center of the lower shaft than the target shape, as presented in Figure 13a.

Further, Figure 13b illustrates the result of the comparative evaluation between the numerically predicted feature and the required geometries of the drive shaft. Altogether, the results showed that the dimensional variations were small and consistent with the range of −0.25 mm to 0.25 mm. However, according to the locally distributed error contour illustrated in Figure 13b, the numerically obtained drive shaft, as compared with the designed shape, had a sinking depth of about −2.47 mm in the upper head region, along with an additional protrusion of 1.03 mm around the center part of the lower shaft and a difference of −1.72 mm at the end of the extruded spur gear section. Remarkably, the dimensional error distributions in Figure 13 differed slightly from the numerically predicted values, as the vertical distance illustrates in Figure 11. However, these variations can be attributed to the fact that the image processing software used in this study displays the relative distances between the comparative objects.

## 6. Conclusions

In this study, a drive shaft that two distinctive features such as the spur gear feature and the internal spline geometry were merged into a single shaft was introduced. In particular, the internal spline structure had the details of an irregular hexadecagonal groove with a depth of about 22.70 mm, while the spur gear geometry had a sixteen-tooth profile with a face width of nearly 92.00 mm. In order to realize the drive shaft by the cold forging operations, and by using the spheroidized and annealed AISI 1035 workpiece and three-dimensional one-sixteen (1/16) FE models with a cyclic planar symmetric condition, a series of parametric investigations related to the shoulder angle of each extrusion die were performed through a series of FEM-based numerical simulations. Based on the results extracted from the FEM-based numerical simulations, compliance evaluations of the dimensional specifications required for the drive shaft were carried out. The results obtained from these parametric investigations can be summarized as follows:

(1) Due to the geometric distinctiveness of the drive shaft in which the internal spline structure and the spur gear feature were positioned almost adjacent to each other, the combined extrusion process was used for the concurrent cold forging. Furthermore, to avoid excessive plastic deformation of the drive shaft, the preform was applied as an intermediate billet.

(2) Through a series of evaluations of the effects of the shoulder angles on the product qualities of the numerically visualized preform and realized drive shaft, it was ensured that the preform and the drive shaft can be felicitously fabricated when the geometric combination of ***θ*_1_** = 45° and ***θ*_2_** = 45° with respect to the shoulder angles was chosen.

(3) In terms of the dimensional relevance, it was verified that the numerical simulation results have a meaningful dimensional accuracy in the range of acceptable variations in view of the main shapes and the required dimensions.

(4) It was conclusively confirmed that the drive shaft, which was composed of the spur gear and the internal spline, can be adequately produced through the combined cold extrusion process proposed in this study.

(5) Based on the results visualized with the numerical simulations and parametric investigations, a series of experimental verifications including the design and fabrication of the cold forging tool components for the prototyping and mass production of the drive shaft will be introduced in the near future.

## Figures and Tables

**Table 1:** Mechanical properties of AISI 1035 medium carbon steel as the cold-drawn round specimen.

Tensile Properties		Raw Material		Annealed Material	
		Engineering	True	Engineering	True
Young’s Modulus (GPa)		196	196	196	196
Yield Strength (MPa)		410	410	350	350
Ultimate Strength (MPa)		621	755	509	643
Poisson’s Ratio		0.29	0.29	0.29	0.29
Fracture Strain		0.32587	-	0.37845	-
Work-Hardening Law, $\bar{\sigma }=K{\bar{\varepsilon }}^{n}$	*K*	-	-	-	900
	*n*	-	-	-	0.225

**Table 2:** Available combinations of the geometric parameters for the preform and drive shaft.

Geometric Parameter	Preform (*θ*_1_)	Drive Shaft (*θ*_2_)	Combinations (*θ*_1_, *θ*_2_)
Shoulder Angle on Extrusion Die (Middle)	30°	30°	Case 1 (30°, 30°)
		45°	Case 2 (30°, 45°)
		60°	Case 3 (30°, 60°)
	45°	30°	Case 4 (45°, 30°)
		45°	Case 5 (45°, 45°)
		60°	Case 6 (45°, 60°)
	60°	30°	Case 7 (60°, 30°)
		45°	Case 8 (60°, 45°)
		60°	Case 9 (60°, 60°)

**Table 3:** Compatibility evaluation of the shoulder angles (***θ*_1_**, ***θ*_2_**) on the extrusion dies (middle).

		Shoulder Angle (*θ*_1_)		
		30°	45°	60°
**Shoulder** **Angle** **(*θ*_2_)**	**30°**	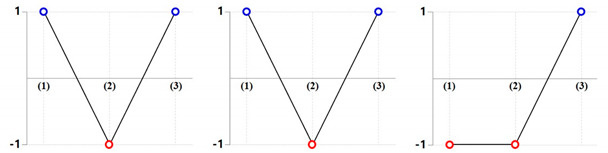		
	**45°**	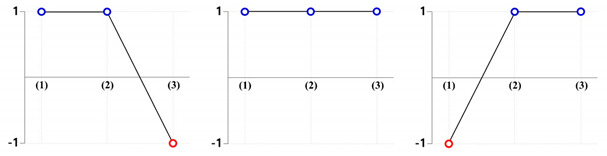		
	**60°**	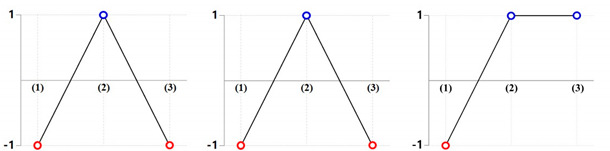		
Checking Criteria		(1) Face width of tooth: 92.00 mm ± 1.50 mm (Satisfy: “**1**”, Fail: “**-1**”) (2) Sinking depth on deep groove: ≤ 2.00mm (Satisfy: “**1**”, Fail: “**-1**”) (3) End shape of extrusion tooth: ≤ 1.00mm (Satisfy: “**1**”, Fail: “**-1**”)		
